# Selective neural stimulation methods improve cycling exercise performance after spinal cord injury: a case series

**DOI:** 10.1186/s12984-021-00912-5

**Published:** 2021-07-23

**Authors:** Kristen Gelenitis, Kevin Foglyano, Lisa Lombardo, Ronald Triolo

**Affiliations:** 1 Case Western Reserve University, 10900 Euclid Ave, Cleveland, OH 44106 USA; 2 Louis Stokes Cleveland VA Medical Center, 10701 East Blvd, Cleveland, OH 44106 USA

**Keywords:** Paralysis, Spinal cord Injury, Exercise, Neural stimulation, Musculoskeletal, Cycling, Fatigue

## Abstract

**Background:**

Exercise after paralysis can help prevent secondary health complications, but achieving adequate exercise volumes and intensities is difficult with loss of motor control. Existing electrical stimulation-driven cycling systems involve the paralyzed musculature but result in rapid force decline and muscle fatigue, limiting their effectiveness. This study explores the effects of selective stimulation patterns delivered through multi-contact nerve cuff electrodes on functional exercise output, with the goal of increasing work performed and power maintained within each bout of exercise.

**Methods:**

Three people with spinal cord injury and implanted stimulation systems performed cycling trials using conventional (S-Max), low overlap (S-Low), low duty cycle (C-Max), and/or combined low overlap and low duty cycle (C-Low) stimulation patterns. Outcome measures include total work (W), end power (P_end_), power fluctuation indices (PFI), charge accumulation (Q), and efficiency (η). Mann–Whitney tests were used for statistical comparisons of W and P_end_ between a selective pattern and S-Max. Welch’s ANOVAs were used to evaluate differences in PFIs among all patterns tested within a participant (n ≥ 90 per stimulation condition).

**Results:**

At least one selective pattern significantly (p < 0.05) increased W and P_end_ over S-Max in each participant. All selective patterns also reduced Q and increased η compared with S-Max for all participants. C-Max significantly (p < 0.01) increased PFI, indicating a decrease in ride smoothness with low duty cycle patterns.

**Conclusions:**

Selective stimulation patterns can increase work performed and power sustained by paralyzed muscles prior to fatigue with increased stimulation efficiency. While still effective, low duty cycle patterns can cause inconsistent power outputs each pedal stroke, but this can be managed by utilizing optimized stimulation levels. Increasing work and sustained power each exercise session has the potential to ultimately improve the physiological benefits of stimulation-driven exercise.

## Background

Upper motor neuron paralysis resulting from spinal cord injury (SCI) and other neuromuscular disorders leads to numerous secondary health complications. Chronic immobility contributes to muscle atrophy, bone density loss, poor circulation and tissue viability, pressure injuries, and cardiovascular disorders that negatively affect survivors’ overall health and quality of life [1]. These complications place survivors at a much greater mortality risk than the general population [2]. Exercise is crucial for mitigating or even preventing some of these secondary health issues and improving long-term prognoses [3], but achieving adequate exercise volumes is difficult with loss of volitional control over a large percentage of total muscle mass [4].

People with lower extremity paralysis have historically been restricted to exercising with only the upper body muscles for which they maintain control. Hand-cycling and arm weight exercises maintain upper body tone and provide some cardiovascular workout, but do not address the complications specific to the paralyzed lower extremities. Electrical stimulation-driven exercise systems provide a more complete, full-body workout by engaging the paralyzed musculature [5, 6]. Specific patterns of electrical impulses activate still-innervated but disconnected muscles below the level of injury to reproduce cycling or rowing exercise motions that can no longer be achieved volitionally [6, 7]. Several studies have shown marked improvements in lower extremity muscle mass [8], body composition [9], and perceived quality of life [10] after participants with SCI train with these systems.

However, bypassing the natural muscle activation pathway with electrical stimulation introduces several complications that limit the observed physiological benefits. As opposed to the natural recruitment order of small, slowly fatiguing fibers first followed by larger, faster fatiguing fibers only as needed [11], extracellular stimulation recruits motor unit pools in a non-selective, spatially fixed manner [12]. Larger, fast-fatiguing fibers may thus be activated and exhausted much sooner than with physiological activation. This problem is compounded by the conversion from Type I (slow twitch, non-fatigable) to Type II (fast twitch, fatigable) that occurs after SCI [13]. Additionally, all fibers reaching firing threshold in response to stimulation will fire synchronously in time with each stimulus pulse [12]. This simultaneity and forced firing frequencies are in stark contrast to the stochastic and adaptive firing patterns observed under physiologic activation, and contribute to quicker contractile force decline in all fiber types. These phenomena ultimately result in rapid muscle fatigue, low sustained exercise intensity, and poor endurance with stimulation-induced exercise systems [5].

Due to these inherent limitations, significant improvements in physiological markers of fitness such as bone density, which is load-dependent and may require longer exercise durations against higher resistances, have not been well reproduced with electrical stimulation systems [14–17]. Many of the significant physiological improvements that have been cited typically come only after extensive long-term training regimens, which may not be a feasible or appealing commitment to most people with paralysis. Furthermore, delayed results and perceived low benefit-to-cost ratio, despite evidence of eventual health improvements with continued use, are commonly cited reasons for abandonment of this technology [18]. In an attempt to extend exercise durations and increase usability, commercially available functional electrical stimulation (FES) bikes have built in motors that assist in pedaling when the muscles are fatigued. However, use of a motor removes much of the resistance training aspect of cycling and instead turns it into a passive range of motion exercise for the user. To yield compelling physiological improvements, the muscles themselves must be solely responsible for producing and sustaining strong exercise outputs. There is thus a need to identify stimulation strategies that prolong activated muscle output and enable individuals with paralysis to achieve longer and more intense workouts each exercise session for maximal results.

One potential way to acutely improve electrically-induced exercise is to reduce the overlap of activated fibers among stimulating electrodes. Current systems stimulate through multiple surface electrode pads [19–22] or implanted neural electrode contacts [23] at once and at high pulse amplitudes (PA) and/or pulse widths (PW) to engage as many muscle fibers as possible, particularly during knee extension phases of cycling. Though this can result in high initial power production, the large voltage fields produced by each electrode can overlap and limit performance as the exercise goes on. Stimulation through multiple electrodes is rarely perfectly synchronized, so motor units within the overlapping regions will be forced to fire at higher frequencies than intended due to the summation of the slightly asynchronous fields. For example, a motor unit within a region of overlapping fields from two electrodes stimulating individually at 20 Hz can experience a combined firing frequency demand of 40 Hz. Higher firing frequencies have been shown to increase rates of fatigue [24], so these overlapping fields likely contribute to the considerable decline in force and power production seen shortly after the onset of stimulation. Using selective, multi-contact nerve cuff electrodes [25–27] and overlap optimization techniques described in [28], stimulation levels can be adjusted through individual electrode contacts to provide ample muscle recruitment with minimal field overlap, which may improve cycling performance.

A second approach that may acutely improve stimulation-driven exercise is to reduce the duty cycle of activated motor units. Conventional cycling stimulation methods activate large groups of synergistic muscle fibers each pedal rotation. For example, large portions or even multiple heads of the quadriceps are activated concurrently when strong knee extension is needed [23]. The activated fibers thus have a high duty cycle, or work to rest ratio, as they are all repeatedly activated each pedal stroke. Studies have shown that high duty cycles contribute to rapid muscle fatigue and force decline, whereas lower duty cycles can extend muscle output prior to fatigue [29–31]. Duty cycle may be lowered without interrupting cycling motion by alternating between muscles with a “carousel” stimulation pattern through selective, multi-contact electrodes [26, 27, 32, 33]. Carousel stimulation rotates activation among multiple independent yet synergistic subsets of fibers such that one performs the desired action while the others rest and recover. By activating only one independent motor unit pool (MUP) each pedal stroke and alternating which pool is active or resting every pedal rotation, duty cycle can be decreased and cycling performance improved.

The goal of this study is to explore the relative effects of low overlap stimulation and low duty cycle stimulation in isolation and in combination to determine their acute effects on cycling performance after SCI. We hypothesize that reducing the overlap and/or duty cycle of activated fiber groups will increase functional work performed within an exercise session over conventional stimulation techniques.

## Methods

### Participants and technology

**Fig. 1:**
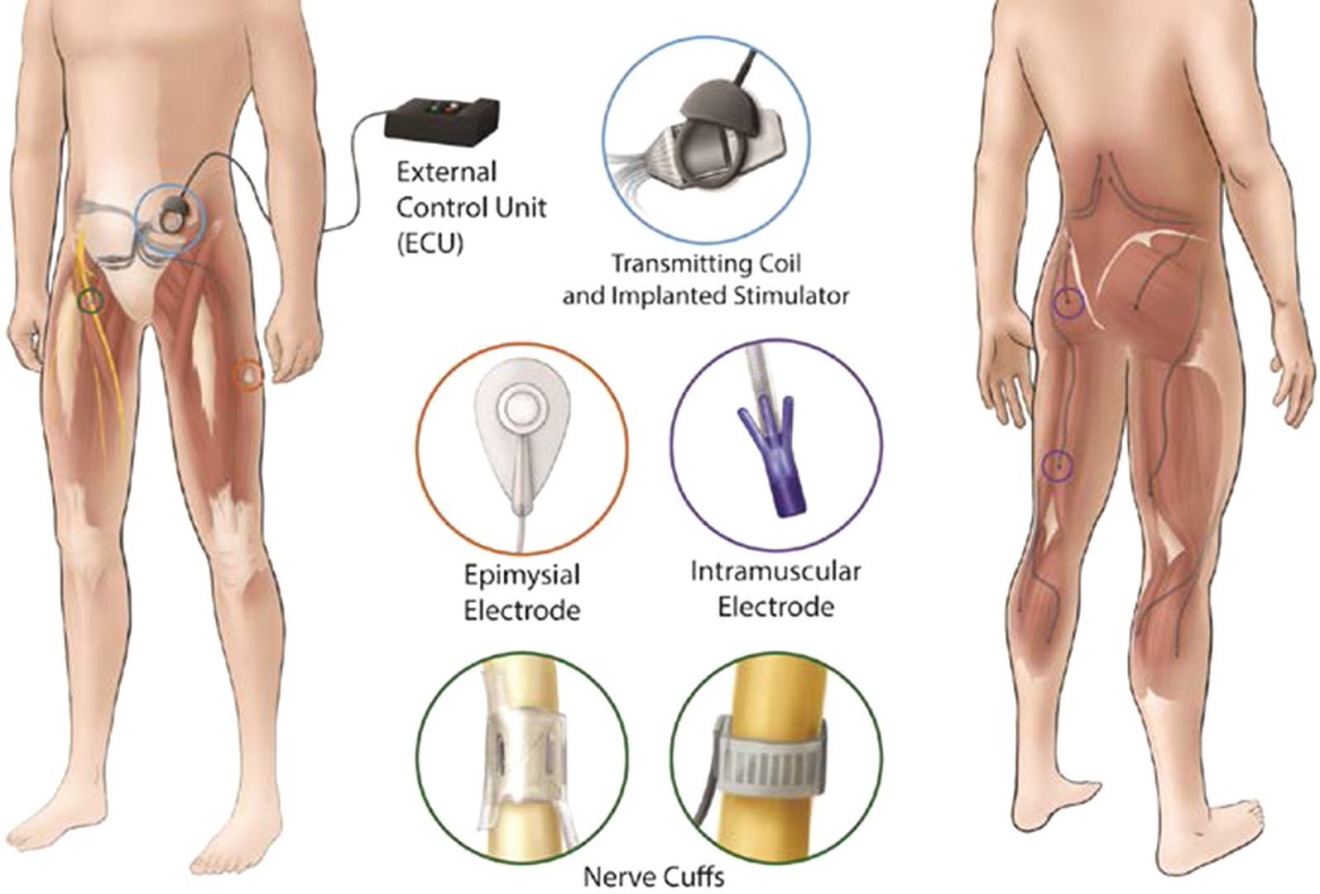
Generalized selective neural stimulation system implantation layout. Multi-contact cuff electrodes are sutured around the femoral nerves to elicit knee extension. Additional intramuscular or epimysial electrodes activate the gluteal and sciatic nerves for hip extension via the gluteus maximus and hamstrings. Electrodes connect to a subcutaneous implanted pulse generator sutured to the abdominal fascia that is powered by and communicates with an external control unit (ECU) via an inductive link with a radiofrequency coil taped to the skin surface. The ECU is programmed with the desired stimulation levels and timing patterns for a particular movement and relays them to the stimulator

**Fig. 2:**
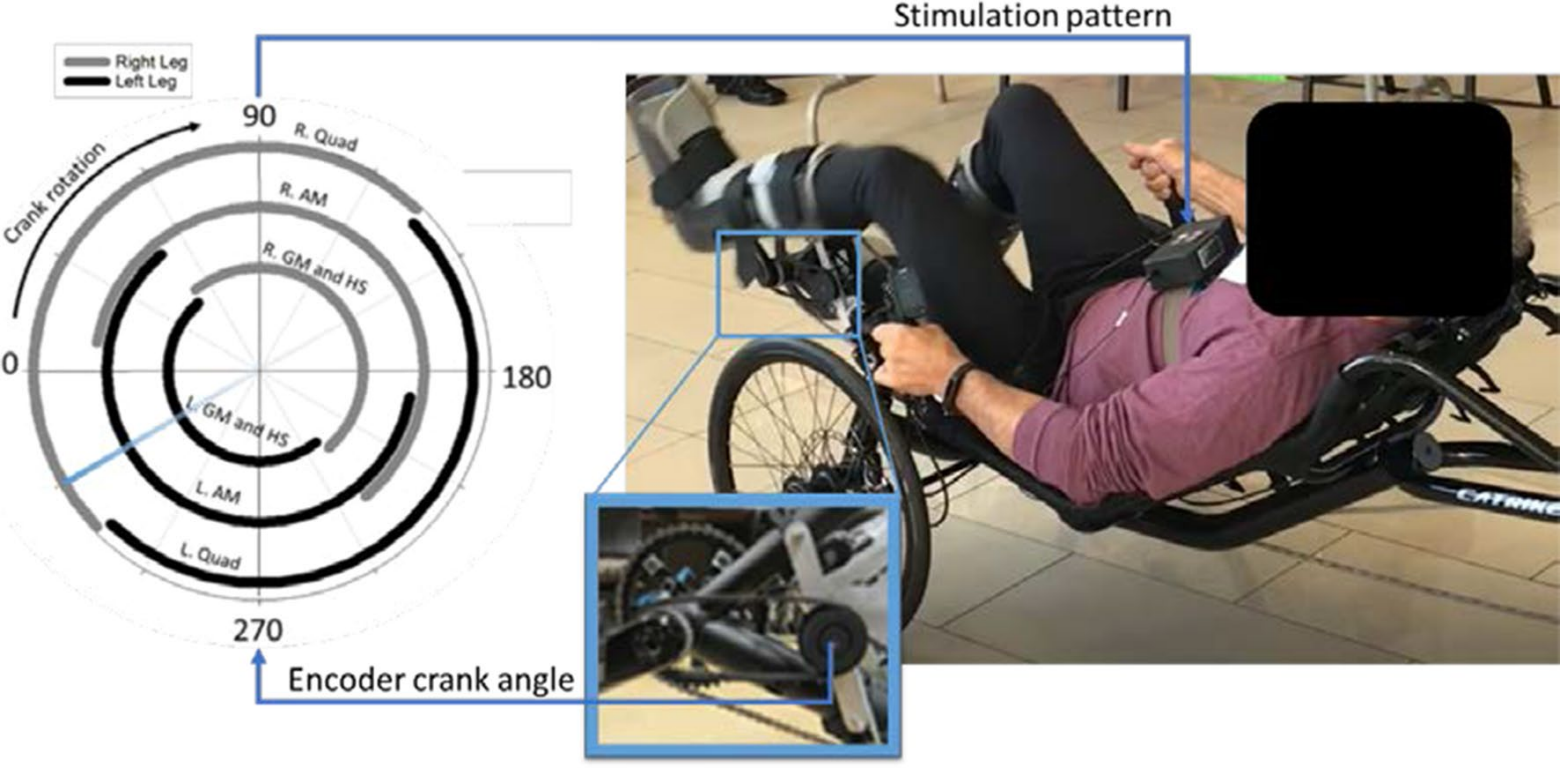
Stimulation-driven cycling exercise setup. Stimulation through implanted electrode contacts is determined by instantaneous pedal crank angle from an encoder on the recumbent bike

Three individuals with SCI with implanted neural stimulation systems (Fig. 1) customized for other studies of standing, stepping or transfers in our laboratory [34–37] participated in the selective stimulation-driven exercise experiments. A crank angle encoder (US Digital, Inc.) relayed instantaneous recumbent bike pedal crank position to an external control unit (ECU) [38] running custom cycling exercise stimulation models as a Simulink real-time xPC target. Crank angle was mapped to the necessary muscle activations and timings for smooth cycling within the ECU stimulation model [23]. The ECU relayed the desired stimulus based on crank angle via a close coupled inductive radiofrequency communications link to a subcutaneous implanted pulse generator. The implanted stimulator then delivered appropriate charge balanced, current controlled, asymmetric, pulse width modulated waveforms through intramuscular or epimysial electrodes near the motor nerves of the desired hip and trunk muscles, or through individual multi-contact nerve cuff electrode contacts on the femoral nerves to activate individual portions of the quadriceps group (Fig. 2). The implanted components of this system have been shown to provide stable longitudinal performance without damage to the stimulated neural tissue [35, 39–41].

The quadriceps, hamstrings, adductors, and gluteal muscles may all be involved in the stimulation patterns to generate cycling exercise. For this study, only activation of the quadriceps (knee extensors) varied among stimulation conditions. In two participants (P02 & P03), composite flat interface nerve electrodes (C-FINEs) [42] were bilaterally implanted around the proximal femoral nerves. Prior studies found three contacts on each C-FINE could selectively activate independent knee extensor MUPs in such cases [43]. The other participant (P01) had bilateral spiral cuff electrodes [35] around the proximal femoral nerves and epimysial electrodes sutured near the motor point of each vastus lateralis (VL). Two contacts per spiral cuff and the epimysial electrode were found to selectively activate independent knee extensors [28]. Each participant therefore had three independently controlled electrical contacts that elicited separate MUPs for knee extension, enabling the study of the effects of overlap and duty cycle. A summary of participant demographics and implanted electrodes of interest to this study is provided in Table 1.

**Table 1:** Participant information and implanted stimulation system descriptions

Participant	SCI Classification	Activity Level (rides per week)	Knee Extensor Stimulation (Independent Contacts)
P01	C7 ASIA-B	High (3–4)	Proximal femoral spiral cuffs (2) VL epimysial (1)
P02	T10 ASIA-A	Moderate (1–2)	Proximal femoral C-FINEs (3)
P03	C7 ASIA-C	Low (< 1)	Proximal femoral C-FINEs (3)

### Cycling protocol and stimulation conditions

**Fig. 3:**
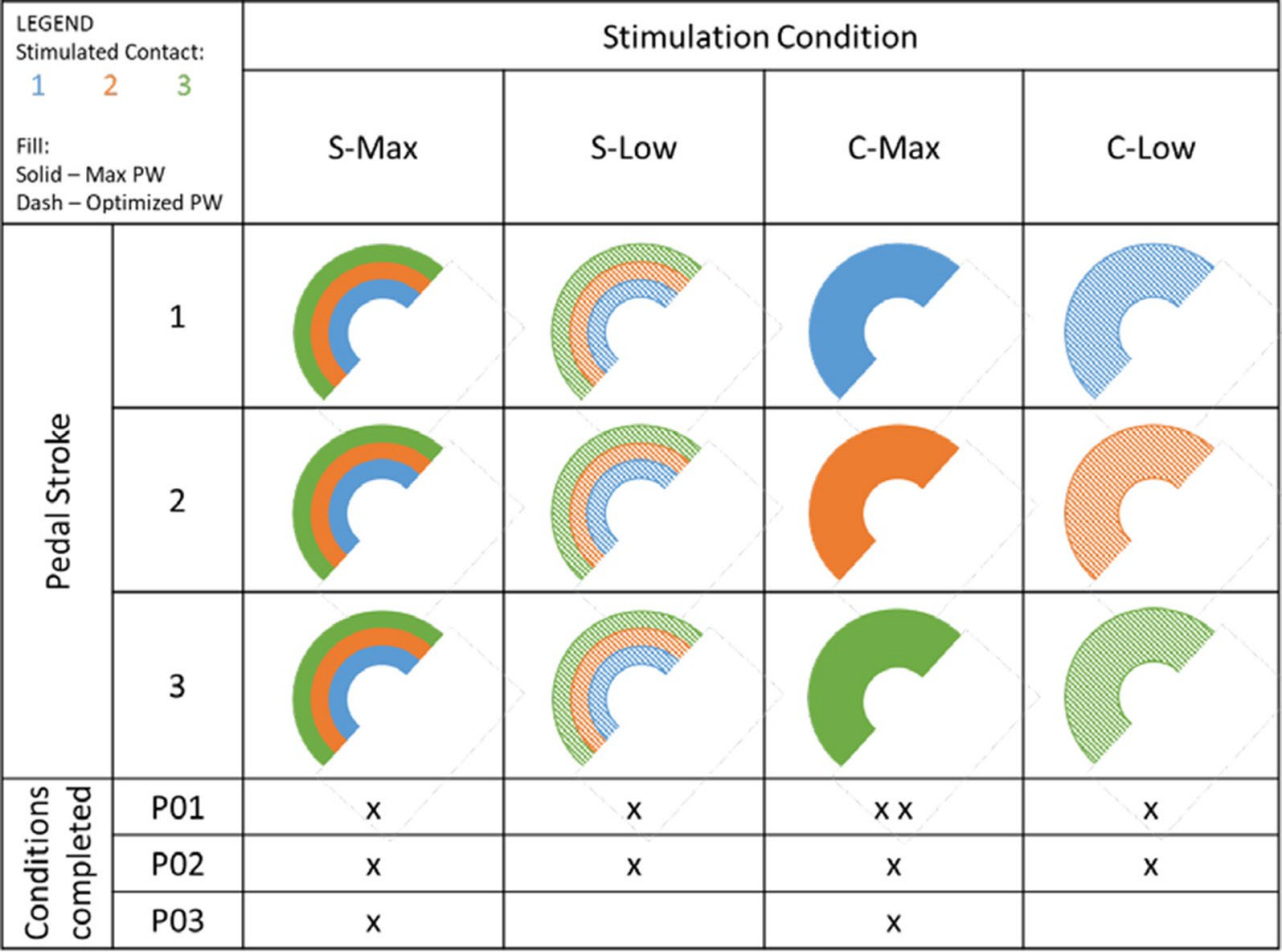
Cycling exercise stimulation conditions completed by each participant. Colors represent the electrical contact(s) delivering stimulating current through each pedal stroke for a single leg and fills represent stimulation level. (1) S-Max is the conventional pattern which activates multiple MUPs each pedal stroke at supramaximal intensities. (2) S-Low activates multiple MUPs each pedal stroke at optimal PWs found through moment summation tests to reduce overlap among activated fibers. (3) C-Max activates one MUP per pedal stroke at supramaximal intensities, rotating active MUP each revolution to reduce duty cycle. (4) C-Low combines low duty cycle and low overlap approaches by rotating activation of a single MUP each pedal stroke and stimulating at optimized PW levels. Note that P01 completed two variations of the C-Max stimulation condition involving different numbers of independent MUPs

While seated in a recumbent tricycle (Catrike, Orlando, FL) with their legs secured in custom orthotic boots secured to the pedals, participants used their arms to manually cycle their legs for approximately one minute to overcome initial tightness and spasticity in the paralyzed musculature. If necessary, additional stretches were performed until manual cycling was easily achieved without excessive resistance from underlying muscle tone. Participants then performed stimulation-induced cycling trials with up to five different stimulation conditions to determine effects of fiber overlap and duty cycle on exercise outcomes (Fig. 3).

The Standard, Maximum Overlap (S-Max) condition represents conventional stimulation. Each knee extensor MUP is supra-maximally activated (Frequency = 25 Hz, PA = 0.8 mA, PW = 255 µs) each pedal rotation, resulting in both high duty cycles and high overlaps among activated MUPs. The Standard, Low Overlap (S-Low) condition similarly activates each knee extensor MUP every pedal rotation, but uses optimized stimulation PW values found through moment summation tests on a dynamometer prior to exercise to reduce overlap among activated fibers. Moment summation tests examined the difference between actual moment output when one MUP is stimulated within the refractory period of another, and ideal summation of the outputs generated when each MUP was stimulated individually. Perfect summation indicated completely independent yet synergistic MUPs. A difference between actual and ideal summation was used to calculate functional percent overlap. Moment summation tests were performed across a wide range of PWs through each involved contact to identify those that generated enough muscle output for a given task while keeping overlap below a chosen threshold. See reference [28] for more details. These optimization procedures generally resulted in submaximal stimulus levels delivered through each involved contact in the S-Low condition.

A summary of the optimized stimulation levels and corresponding decreases in overlap compared with conventional stimulation is provided in Table 2. P03 did not complete overlap testing sessions due to time limitations and upper extremity muscle injury unrelated to this study, and so did not cycle with test conditions involving overlap reduction. P01 and P02 cycled with the listed stimulation parameters during all test conditions involving low overlap. Note that stimulation through one contact in each leg of P01 resulted in high overlap with other contacts with little additional muscle output at output levels required for cycling, so it was excluded from the low overlap stimulation pattern.

**Table 2:** Stimulation parameters used during low overlap test conditions and the resulting overlap reductions, presented as the mean ± standard deviation of the difference in overlap between optimized and maximum stimulation levels for each pairwise combination of contacts

Participant	Leg	Low Overlap Stimulus Level [us]			Overlap Reduction
		Contact 1	Contact 2	Contact 3	
P01	Right	175	105	0	21.2 ± 3.2%
	Left	105	0	95	30.4 ± 1.1%
P02	Right	165	160	150	16.8 ± 5.9%
	Left	145	105	100	18.8 ± 8.0%

Carousel with Maximum Overlap (C-Max) rotates supramaximal stimuli (PW = 255 µs) through independent contacts to maximally activate a different MUP each pedal stroke, reducing duty cycle. P01 performed carousel cycling trials involving all three contacts (C-Max 3c) as well as just the two strongest contacts per leg (C-Max 2c), as one activated fiber pool per leg was significantly weaker than the others. This enabled insight into the trade-off between keeping duty cycle as low as possible by including a weak group and having less duty cycle reduction but only relying on the strongest fibers. Following observations from P01, carousel stimulation trials with the remaining participants were set to involve only their strongest MUPs. Therefore, for P02, three MUPs on the right leg and only the two strongest MUPs on the left leg were used. For P03, only the two strongest MUPs on each leg were used. Carousel, Low Overlap (C-Low) combines low overlap and low duty cycle approaches by activating one MUP per pedal rotation with optimized stimulus values.

Each experimental session began with a warm-up trial with stimulation followed by a S-Max stimulation trial to obtain baseline performance metrics with their usual cycling pattern. Subsequent trials alternated stimulation conditions between one of the test conditions and S-Max. Exercise trial durations were determined based on the amount of time the participant could continuously cycle with the conventional S-Max stimulation pattern (5, 2, and 1.5 min for P01, P02, and P03 respectively). Around their respective maximal trial times, P02 and P03 consistently paused at various points of the pedal stroke and needed to push their leg with their arm to continue, prompting cutoff at those durations. P01 could cycle well beyond 5 min, but time limitations prompted us to end trials when power output typically reached a steady state. Though trial durations varied by participants based on ability level, trial durations were kept consistent across simulation conditions within subjects. To prevent any cumulative effects of fatigue from influencing the results, rest breaks at least double each participant’s respective active cycling times (i.e., at least 10, 4, and 3 min for P01, P02, and P03 respectively) were imposed between trials.

### Outcome measures and statistical analysis

Cycling performance metrics were measured by a Garmin Edge bike computer (Garmin Ltd., Olathe, KS) communicating with Quarq DZero power crank arms (SRAM LLC, Chicago, IL). Total work (W) was calculated as cycling power output integrated over trial duration. Increased W indicates greater exercise intensity was maintained throughout the trial, as trial durations were equal across stimulation conditions within each participant. End power (P_end_) averaged the power output over the final third of each trial to approximate steady state power output. Higher P_end_ indicates that a stimulation condition improves steady state power maintenance. W and P_end_ are presented as the differences in work and end power (ΔW and ΔP_end_ respectively) between test condition and S-Max trials from the same experimental session. A power fluctuation index (PFI) was calculated over each 6 s window to encompass several full pedal revolutions to characterize the smoothness of power production between pedal strokes for each stimulation condition. To calculate PFI, a linear least squares line was fit to the raw power data within each window to establish a local trend. The maximum and minimum difference between raw power and the fitted line were used to calculate a power deviation range around the general trend in each window. This range was then divided by the average of the trend for the PFI of that window. Examining the fitted trend within each window ensures steady decreases in power due to fatigue are not interpreted as large differences between pedal strokes. Lower PFIs indicate more consistent power outputs and smoother rides. Charge accumulation (Q) was calculated as the integral of the pulse amplitude multiplied by pulse width over time to characterize differences in stimulation efficiency (Δη) between S-Max and test conditions:

$$\Delta \eta = \frac{W}{Q}- \frac{{W}_{S-Max}}{{Q}_{S-Max}}$$

Each participant performed a minimum of six trials per test condition, each with a corresponding number of conventional stimulation trials for comparison. Homogenous (Levene’s test for equal variances p > 0.05), though sometimes non-normal (Shapiro-Wilks test for normality p < 0.05) W and P_end_ data sets prompted application of Mann–Whitney tests for nonparametric comparison of two independent samples (test condition trial outcomes vs. corresponding conventional stimulation trial outcomes). PFI data sets were found to be neither homogenous nor normally distributed. PFI were thus compared among all stimulation conditions using Welch’s ANOVAs followed by post-hoc Dunn’s tests using Matlab’s *multcompare* function. Welch’s ANOVAs do not assume equal variances and are robust to deviations in normality with sufficient sample size (n > 180 for all conditions and participants).

## Results

### Total work and end power

**Fig. 4:**
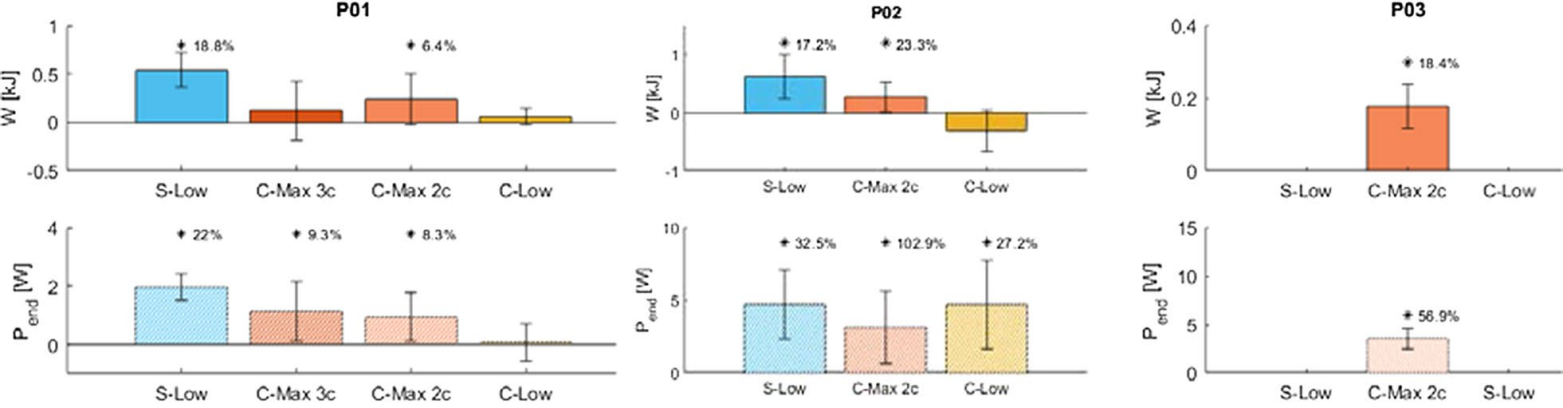
Differences in W and P_end_ for each test condition compared with S-Max stimulation for P01 (left), P02 (middle) and P03 (right). Percent increase is listed for differences with statistical significance (p < 0.05)

W and P_end_ were examined as the differences in outcomes from trials of a given test condition and S-Max trials from the same experimental session and are shown in Fig. 4. Positive ΔW values indicate that the test condition enabled more work to be performed than conventional stimulation in the same amount of time. Positive ΔP_end_ values indicate a test condition increased steady state power maintenance over conventional stimulation. Significant positive ΔW and ΔP_end_ were found for various test conditions in all participants. For conditions with statistical significance, percent improvement relative to S-Max outcomes are also included in Fig. 4.

P01 performed significantly more work after 5 min of cycling with S-Low (18.8%, p < 0.05) and C-Max 2c (6.4%, p < 0.05) stimulation than with conventional (S-Max) stimulation. P01 also showed a trend of slightly increased W with C-Max 3c and C-Low, though these differences were not significant. Except for the C-Low condition, all test conditions maintained significantly higher end powers than conventional stimulation (22.0%, 9.3%, and 8.3% for S-Low, C-Max 3c, and C-Max 2c respectively, p < 0.05).

P02 performed significantly more work than S-Max after 2.5 min of cycling with S-Low stimulation (17.2%, p < 0.05) and C-Max 2c (23.3%, p < 0.05). W decreased with C-Low stimulation, though not significantly. P_end_ was significantly higher than S-Max for all test conditions (S-Low 32.5%, C-Max 2c 102.9%, and C-Low 27.2%, p < 0.05). Note that percent improvements were greater for C-Max 2c despite the absolute value of the differences being lower than with the other stimulation methods. This was due to a large decrease in baseline cycling ability from an extended period of inactivity during the COVID-19 pandemic before C-Max 2c could be tested. Thus, while the absolute value of W and P_end_ improvements were smaller when using the C-Max 2c pattern, they actually reflected larger improvements relative to conventional stimulation trials performed at that same point in time.

P03 performed significantly more work (18.4%, p < 0.05) with C-Max 2c stimulation after 1.5 min of cycling compared with S-Max stimulation. End Power was also significantly greater (56.9%, p < 0.05) with C-Max 2c stimulation.

### Power fluctuation

**Fig. 5:**
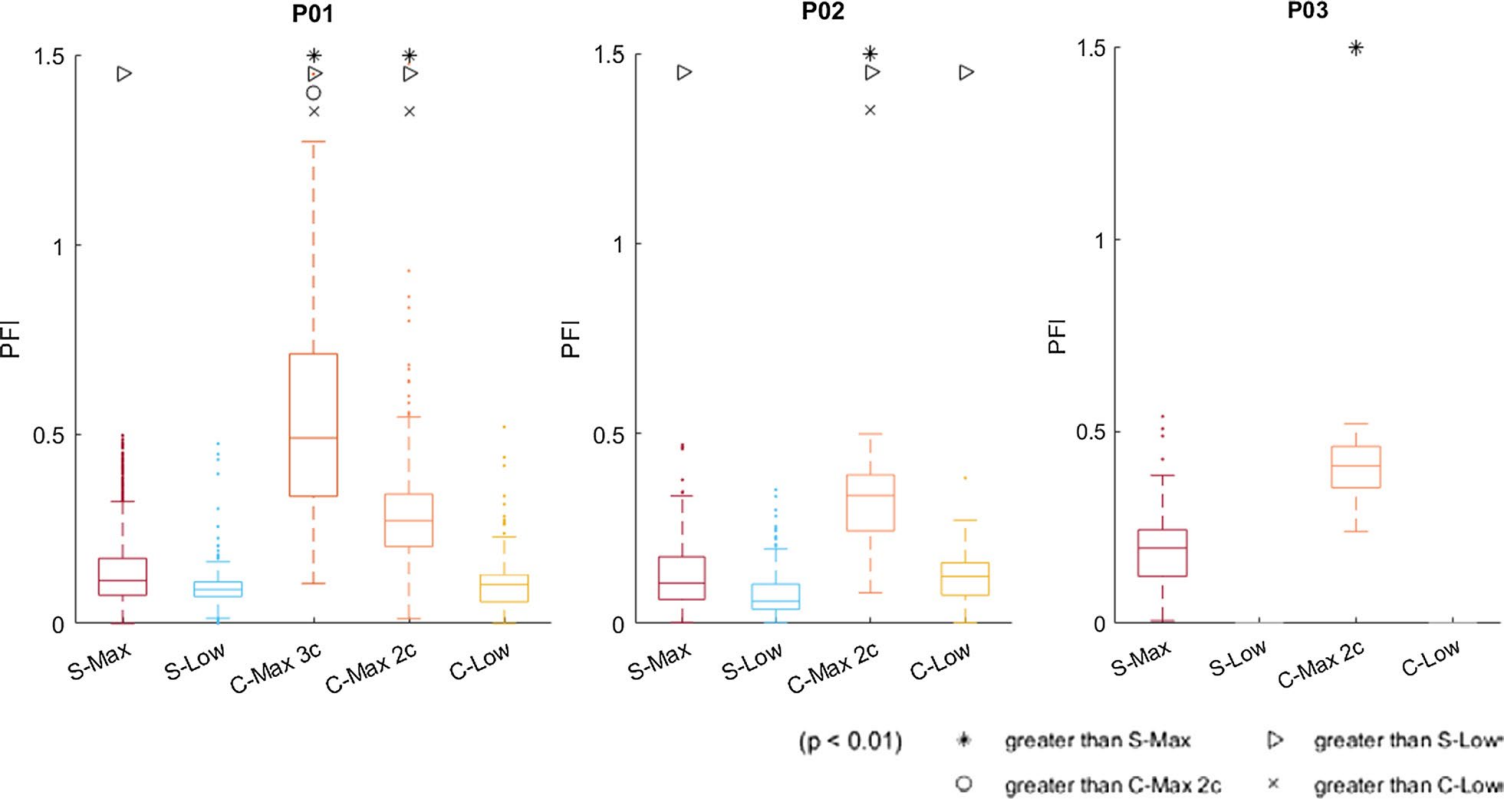
Power fluctuation indices of each stimulation condition for P01, P02, and P03. Lower PFI values indicate lower stroke-to-stroke variability in power output while pedaling and a smoother ride

PFI varied among stimulation conditions (Fig. 5). In all participants, conventional S-Max stimulation resulted in a median PFI below 0.2, meaning less than 20% fluctuation in power typically occurred over several revolutions. S-Low caused a significant PFI decrease relative to S-Max in P01; in P02 no significant difference was found. C-Max patterns with all three contacts increased PFI significantly (p < 0.01) compared with S-Max stimulation in P01, resulting in a PFI median of 0.49 and maximum of 2.7. C-Max with only the two strongest contacts significantly reduced PFI to a median of 0.27 and a maximum of 1.4 in P01, but this was still significantly greater than the S-Max condition. C-Max involving only the strongest contacts similarly resulted in PFIs significantly higher than S-Max with medians of 0.33 and 0.41 for P02 and P03 respectively. C-Low increased PFI slightly relative to S-Max in P02, while in P01 no significant difference was found.

### Charge accumulation and efficiency

**Fig. 6:**
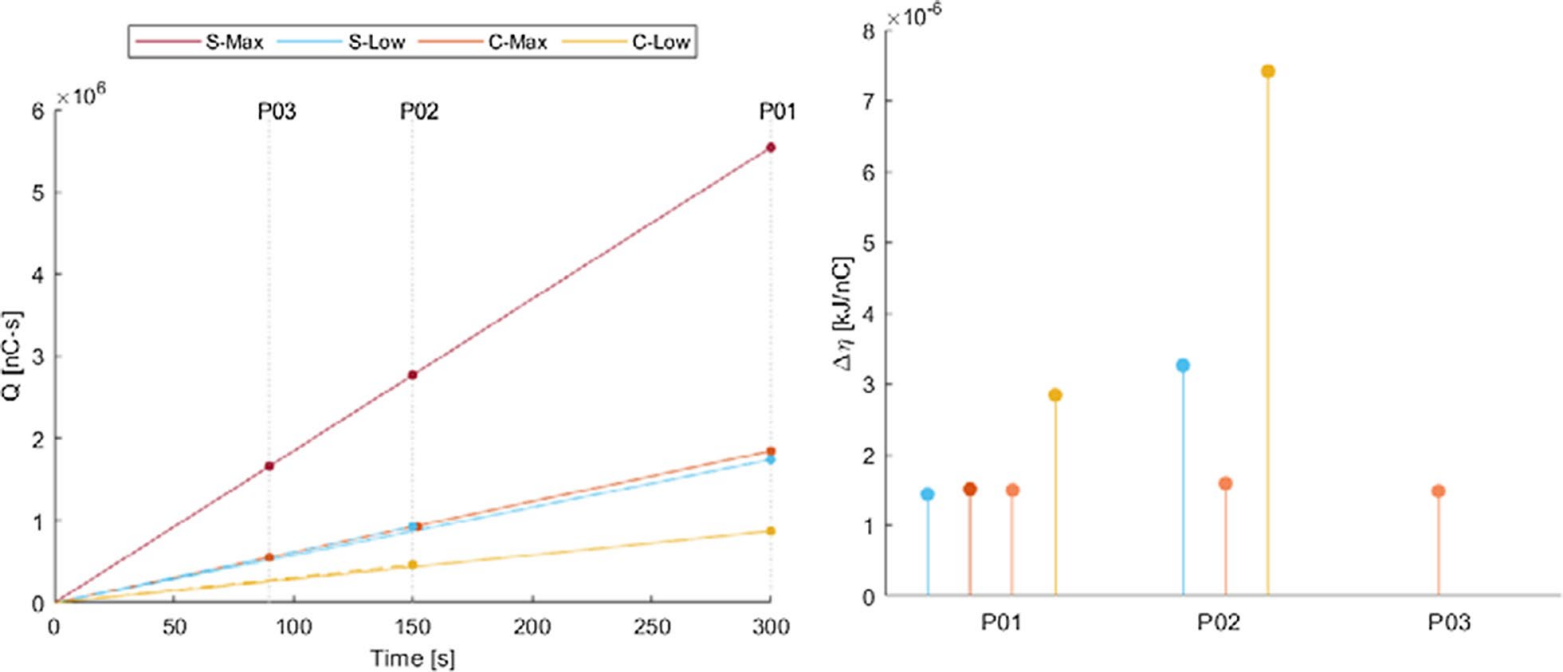
(LEFT) Charge accumulation over time for each stimulation condition. Dots represent total charge injection at the end of each participant’s trial length, indicated by the vertical dotted lines. Low overlap and/or low duty cycle test conditions inject much lower Q than conventional stimulation. (RIGHT) Difference in stimulation efficiency compared with S-Max for each test condition and participant. Positive efficiency differences indicate selective patterns resulted in more work per unit of charge injected

All selective stimulation conditions injected less charge throughout the cycling trials than the S-Max pattern (Fig. 6). C-Low has the lowest Q accumulation as it combined both low overlap and low duty cycle stimulation approaches. S-Low and C-Max had similar Q accumulations that, while higher than C-Low, are still considerably lower than S-Max. All test conditions in all participants resulted in positive Δη, producing more work per unit of charge than with conventional stimulation. C-Low, despite producing insignificant changes in work compared with S-Max, resulted in the highest efficiencies due to the pattern’s extremely low Q (Fig. 6).

## Discussion

### General outcomes

For all participants, at least one stimulation condition significantly improved functional outcome measures over conventional stimulation, demonstrating that selective neural stimulation methods can improve exercise performance after SCI. Though differences exist among participants and patterns, a general discussion for trends in each outcome measure and their implications is first presented in the following sections:

#### Total work

S-Low and C-Max 2c paradigms significantly increased total work performed over conventional (S-Max) stimulation, effectively enabling a more intense workout within the same amount of time. Studies have shown that cycling intensity, rather than duration, has a significantly greater influence on predictors of future health [44], and that higher intensity cycling workloads result in greater improvements in leg strength, body composition, cholesterol levels, and blood pressure compared with longer exercises at low intensities [45]. Furthermore, enabling participants with SCI to achieve more work in less time may improve exercise regimen satisfaction and adherence.

Unlike the other test conditions, C-Low did not result in significantly different work compared with conventional stimulation. In participant P02, C-Low seemed to decrease work performed in the same amount of time, though not significantly. These results are unsurprising as C-Low delivers the lowest amount of stimulation to the fewest MUPs out of all the patterns tested. The peak power produced at the beginning of C-Low trials was often much lower than with other stimulation patterns, causing work, the integral of power over time, to accumulate much more slowly. C-Low should not be eliminated as a viable stimulation option, though, because it provides other benefits discussed below.

#### End power

S-Low and C-Max patterns also significantly increased end power, suggesting they could continue to outperform conventional stimulation in longer cycling trials. Even C-Low, which did not produce significant increases in work, still resulted in significantly higher end power in one participant. In this case, significant work increases may have been seen if trial durations were sufficiently long. Trial durations were limited in this study due to time constraints and the desire to prevent irrecoverable fatigue within a single session, but could be readily extended in future work. Higher end powers also indicate that more force was produced and maintained by the muscles. Greater force production can enable cycling against higher resistances, which may further accelerate strength gains and enable more consistent improvements in load-dependent biomarkers, such as bone density, with these systems [46, 47].

#### Power fluctuation

The power fluctuation index represents the smoothness of a cycling stimulation pattern and is an important consideration for balanced muscle loading and user comfort. No significant difference in PFI was found between S-Low and S-Max in P02, and S-Low significantly decreased PFI relative to S-Max in P01. These results show that cycling smoothness is not compromised by overlap reduction, and in some cases may even be improved. This improvement may be attributed to more balanced force output among the left and right legs at optimized stimulation levels.

C-Max patterns did result in significantly increased PFI and thus greater instability in all participants. This was anticipated since carousel cycling, or any pattern that involves unsynchronized activation of multiple MUPs, risks uneven force production among the different fiber groups. At maximum stimulation levels, the fiber groups activated by the different electrode contacts produced a wide range of power outputs that resulted in some quick and strong revolutions interspersed with slower, weaker ones. The difference in power output among revolutions is reflected in the higher PFI values for C-Max conditions and manifests as a somewhat erratic, jerky cycling motion. This choppiness was most prevalent at the beginning of the carousel trials but subsided as each MUP eventually fatigued, presumably to more consistent levels. For P01, the C-Max pattern involving all three contacts (C-Max 3c) caused occasional pauses in cycling as one fiber group per leg was substantially weaker than the others even when maximally activated. For that reason, C-Max with only the two strongest contacts per leg (C-Max 2c) was also studied. This modified C-Max pattern significantly reduced PFI compared with the three contact version. The increased stability and elimination of pauses in pedaling likely contributed to the significant increase in work with C-Max 2c but not C-Max 3c, as carousel stimulation is always limited by the weakest muscle group activated. There appears to be a clear trade-off between reducing duty cycle to prolong output as much as possible and reducing the overall strength and stability of the pattern as a whole. From these results, it appears that a slightly higher duty cycle with significantly less power fluctuation outperforms the lowest possible duty cycle that exhibits substantial instability. PFI is thus an important area of improvement for carousel cycling so that users may take full advantage of the benefits of low duty cycle without large power variations. Using only the strongest MUPs within the carousel pattern still produced significantly higher PFIs compared to conventional stimulation in all participants, but these power fluctuations were not great enough to make the pattern unusable or be perceived as uncomfortable or distracting.

The C-Low pattern reduced PFI significantly relative to C-Max, even to the point of not being significantly different than S-Max in one participant. This improvement is again likely attributed to more balanced output among fiber groups at optimized stimulation values. However, the powers produced by the few fibers recruited each revolution, while balanced, could not reach nearly the same peak powers as conventional stimulation and often resulted in consistently smooth but weak cycling. Future studies should look to control each contact’s stimulation level throughout a trial to maintain a stable yet strong target power. This would ensure the high stability benefits of C-Low cycling while also gradually recruiting more fibers as necessary to achieve the improved work benefits of C-Max.

#### Charge injection and efficiency

Selective stimulation patterns, by design, inject less charge over time because of their optimized stimulus levels and/or reduced duty cycle. S-Low and C-Max patterns produced more work with less charge than conventional stimulation, and therefore also had higher efficiencies. Even C-Low, which did not differ significantly in work from S-Max, still resulted in more efficient stimulation as it utilized both low overlap and low duty cycle strategies for very low charge injection. Higher efficiency can prolong battery life of the stimulation control units, which is extremely important for practical, everyday use of these systems. Furthermore, reducing the amount of charge needed to produce a desired exercise or motion can greatly decrease the risk of overstimulating and thus minimize any potential of damaging the neural tissue over time [48].

### Pattern-specific discussion

Pattern-specific considerations and differences in performance among participants are addressed in the following sections.

#### Low overlap stimulation

S-Low stimulation-induced cycling was successful for both participants who underwent the stimulation level optimization procedures. Overlap reductions of at least 16% significantly increased total work and end power. In fact, these overlap reductions resulted in some of the largest percentage increases in work performed over conventional stimulation out of all patterns tested in both participants. Further reductions in overlap could extend these advantages, though care must be taken not to drastically reduce stimulation levels to minimize overlap at the expense of adequate muscle output.

Even prior to tuning the system pulse parameters to minimize motor unit overlap in this study, the three out of eight possible femoral nerve C-FINE contacts had been chosen specifically for P02’s conventional stimulation patterns due to their high selectivity for independent knee extensor muscles. Improvements in P02’s performance with S-Low stimulation thus indicates that even already highly selective systems can still be further improved with overlap optimization. P01 has a slightly different stimulation system which is thought to be inherently less selective as there are fewer, larger contacts within the spiral nerve cuff electrodes that may more readily activate the same neural fibers. This is reflected in the fact that one contact per leg was left out of the pattern during optimization, and that the pulse width values needed for low overlap in P01 were considerably lower than those used for P02. Because of the greater initial overlap with conventional stimulation, a greater percent reduction was able to be achieved for P01 than P02, which corresponded with an even greater percent improvement in work. Though this case series does not attempt to make direct comparisons between subjects due to differing ability levels and stimulation systems, the data do support the idea that greater reductions in overlap could result in greater improvements in functional outcome. It also highlights the particular importance of reducing overlap in stimulation systems that do not have a high initial degree of selectivity.

#### Low duty cycle stimulation

C-Max cycling results agree with other studies of similar duty cycle reduction techniques to improve functional outcomes during isometric contractions [29–31, 49, 50]. Improvements with duty cycle reduction are often partially credited to the pumping action that is created when activation is rotated among different fiber groups, which can promote blood flow and oxygen delivery to the muscle [51]. However, as this exercise is cyclic in nature and already comprised of on–off activation patterns within each leg that promote blood flow, it is likely not the main contributor to the success of the carousel stimulation pattern. The carousel stimulation scheme activates each fiber group less often, which can delay glycogen store depletion. The longer rest periods each fiber group experiences can also encourage more complete clearance of metabolite build-up prior to the next contraction. Together, those two benefits may be more likely to account for improved work and power maintenance with C-Max stimulation-induced cycling.

To the authors’ knowledge, only one other study has used a carousel stimulation scheme during a functional cycling task for people with paralysis [52]. That study alternated activation between the rectus femoris and medial/lateral vasti muscles several times within each pedal stroke, as opposed to the one fiber pool per pedal stroke in this study. It demonstrated that an alternating stimulation strategy enabled rides of longer duration and distance while maintaining the same mechanomyogram amplitude in each muscle group. However, no direct power or work measurements were reported and no power fluctuation analyses were included. Furthermore, that study employed two different sized surface electrodes with slightly different placements between the standard and alternating conditions, and did not conduct preparatory overlap analyses to ensure separate fiber populations were being activated in each case. This creates uncertainty as to whether the reported endurance gains were from activation of a different muscle mass or truly from the reduction of duty cycle. In our study, implanted electrodes ensured that the relationship between the contacts and the nerve fibers remained consistent between conditions such that the only changing variable was the stimulation pattern. A priori, overlap analyses also ensured the selectivity of independent motor unit pools, and power fluctuation analysis provided insight into the effects that stimulus modulation strategies have on ride smoothness and user comfort. Our study was clearly able to demonstrate that activating the exact same groups of independent fibers at a lower duty cycle is sufficient to significantly increase sustained exercise intensity.

One suggested advantage of more frequent alternations in the study by Decker et al. [52] is that only the first fiber group activated for each pedal stoke must overcome the passive resistance of the muscle and tendon, thus requiring less effort from the subsequently activated fibers within that revolution. Future studies with implanted electrodes may look to similarly alternate stimulation among multiple contacts within each pedal stroke to take advantage of this. By overcoming the initial resistance to movement with the strongest fiber pool, weaker fiber pools may be able to finish the pedal stroke with minimal effort. This could enable fiber pools that are too weak to effectively cycle on their own, like one observed in each leg of P01 and P03, to effectively contribute to cycling and further improve W and P_end_ measures. However, switching stimulation through various contacts within a pedal stroke may cause unwanted increases in instability and fiber overlap. This is because the sequential ramping up and down of stimulation between contacts during one pedal stroke would have to overlay in such a way to prevent dead spots and pauses. This could cause some fiber groups to be activated before another has fully relaxed, leading to sudden leg jerks and potentially contributing to fiber overlap while two contacts are briefly delivering stimulation at the same time.

#### Combined low overlap, low duty cycle stimulation

C-Low stimulation results varied the most out of all patterns tested. Average work increased in P01 and decreased in P02, though neither were significant. End Power was significantly increased in P02 and largely unchanged in P01. It is possible that C-Low’s success during cycling depends on a participant’s strength and absolute power output capabilities. P02 had power output peaks over four times that of P01 during conventional cycling (80 watts vs 18 watts) but had much lower endurance (2.5 vs 5 min continuous cycling times) due to rapid power decline. C-Low stimulation enabled P02 to cycle approximately 5 watts higher at steady state but achieved a considerably lower peak power compared with conventional stimulation, resulting in significantly higher end power but not total work. With extended trial durations, participants with higher power outputs like P02 and P03 could benefit from C-Low as substantial power is still possible even with the small fiber activation typical of a fatigued state. Participants with much lower power outputs, like P01, may be unable to extend trial durations to the point of significant work improvements as the few fibers activated likely would not produce enough power over time to continue cycling. C-Low may thus be better suited as a short strength building exercise pattern for these weaker participants to specifically target and strengthen individual fiber pools.

## Limitations

No physiological or metabolic measures were recorded in this study as we sought to improve acute performance over conventional stimulation rather than track long-term training results. However, it is well established that higher intensity workouts can lead to greater physiological improvements over time [45]. All cycling trials were performed on an indoor trainer, so these results remain to be verified during over-ground or outdoor use. No official subjective measures of participant preferences between patterns or standardized measures of changes in perceived effort were recorded, though P01 did choose to continue training with the S-Low and C-Max 2c patterns upon study completion. This study also only compares differences in outcome measures between a test condition and conventional stimulation trials performed on that same day. This was done because testing occurred across multiple experimental sessions separated by various time gaps based on participant availability. We did not want training effects from home cycling regimens or fatigue effects from cycling performed earlier in the same week to confound the results, so we always compared test conditions to the S-Max baseline from that same session. This prevented us from drawing a definitive conclusion about which selective stimulation condition (S-Low, C-Max, or C-Low) is superior, but provided certainty as to how a condition compares to the standard pattern under the same circumstances. Finally, it is possible that despite our best efforts and long breaks between trials, some undetected fatigue effects occurred within a session. Because of this, we alternated test conditions with the conventional stimulation pattern, and always presented the conventional trials first. This should ensure muscles are less fatigued during S-Max trials than the test conditions, and strengthens confidence in the positive results observed in spite of having a higher probability of accumulated fatigue.

## Conclusions

The purpose of this study was to implement selective neural stimulation-driven cycling strategies to improve exercise performance in people with lower extremity paralysis. Conventional stimulation systems induce rapid fatigue of the activated musculature, leading to low sustained intensities and slow development of physiological gains. Results of this study show that low fiber overlap and low duty cycle stimulation paradigms can significantly increase work performed during an exercise session over conventional stimulation, and often have potential for further improvement with extended cycling durations, supporting our initial hypothesis. Increasing muscular work performed each exercise session has the potential to ultimately improve physiological outcomes, participant satisfaction, and exercise regimen adherence. Selective stimulation strategies also use less charge over time, improving efficiency of stimulation-induced exercise systems.

Low duty cycle stimulation patterns can cause inconsistent power output among activated fiber groups, reducing ride smoothness and stability, but this can be mediated with optimized stimulation levels and informed electrode inclusion criteria. Inter-subject variability resulted in varying degrees of success for each selective stimulation pattern among participants. Participants with more powerful stimulated responses may benefit from either low overlap or low duty cycle, or even a combination of strategies. Participants with weaker baseline responses may instead benefit most from reductions in overlap until strength is improved, as low duty cycle cycling is greatly affected by the weakest fiber groups. Future work should aim to combine the benefits of low overlap and low duty cycle stimulation through real-time control of carousel stimulation levels. Future work should also incorporate these selective patterns into long-term training regimens and evaluate their direct impacts over time on physiological measures like muscle thickness and metabolic efficiency, as well as their possible indirect impacts on bladder, bowel, and sexual functioning. Finally, the authors recognize that the conventional stimulation strategy is most widely-used because of its simplicity and its ability to be implemented with commercially-available stimulation systems that are limited in channel numbers and in the selectivity of non-invasive surface electrodes. While conventional (S-Max) strategies with surface stimulation are valuable and can provide significant health benefits to users with SCI, such cycling systems often rely on motorized assistance due to low sustained power outputs. Considering the significant potential improvements in power found here, it is our hope that this work encourages expanded use of selective stimulation strategies with either implanted or surface stimulation to eventually increase the intensity of stimulated exercises and ultimately enable routine over ground cycling.

## Data Availability

The datasets analyzed in the current study are available from the corresponding author on reasonable request. Direct correspondence to kxg277@case.edu.

## Abbreviations

SCI: Spinal cord injury
FES: Functional electrical stimulation
MUP: Motor unit pool
ECU: External Control Unit
C-FINE: Composite flat interface nerve electrode
VL: Vastus lateralis
S-Max: Standard maximum overlap stimulation
S-Low: Standard low overlap stimulation
C-Max: Carousel maximum overlap stimulation
C-Low: Carousel low overlap stimulation
W: Work
P_end_: End Power
PFI: Power fluctuation index
Q: Charge
η: Efficiency
